# *Platonia insignis* Mart., a Brazilian Amazonian Plant: The Stem Barks Extract and Its Main Constituent Lupeol Exert Antileishmanial Effects Involving Macrophages Activation

**DOI:** 10.1155/2017/3126458

**Published:** 2017-08-09

**Authors:** Adriana Cunha Souza, Michel Muálem de Moraes Alves, Lucas Moreira Brito, Luciano Gomes De Castro Oliveira, Enoque Pereira Costa Sobrinho-Júnior, Isabella Cristhina Gonçalves Costa, Sâmya Danielle Lima Freitas, Klinger Antonio da Franca Rodrigues, Mariana Helena Chaves, Daniel Dias Rufino Arcanjo, Fernando Aécio de Amorim Carvalho

**Affiliations:** ^1^Medicinal Plants Research Center, Federal University of Piauí, SG-15, Ininga, 64049-550 Teresina, PI, Brazil; ^2^Laboratory of Natural Products, Department of Chemistry, Federal University of Piauí, SG-02, Ininga, 64049-550 Teresina, PI, Brazil; ^3^Department of Biophysics and Pharmacology, Federal University of Rio Grande do Norte, Natal, RN, Brazil

## Abstract

*Platonia insignis* Mart., popularly known as “bacurizeiro,” is used in traditional medical practices based on its diverse biological properties. This study was aimed at evaluating the antileishmanial effects of the ethanol extract (EtOH-Ext), hexane fraction (Hex-F), and its main isolated Lupeol obtained from stem barks of* P. insignis* against* Leishmania (Leishmania) amazonensis*, as well as their cytotoxicity and possible mechanisms of action. The EtOH-Ext, Hex-F, and Lupeol inhibited the growth of* L. amazonensis *promastigote forms at IC_50_ of 174.24, 45.23, and 39.06 *µ*g/mL, respectively, as well as* L. amazonensis* axenic amastigote forms at IC_50_ of 40.58, 35.87, and 44.10 *µ*g/mL, respectively. The mean cytotoxic concentrations for macrophages (CC_50_) were higher than those for amastigotes (341.95, 71.65, and 144.0 *µ*g/mL, resp.), indicating a selective cytotoxicity towards the parasite rather than the macrophages. Interestingly, all treatments promoted antileishmanial effect against macrophage-internalized amastigotes at concentrations lower than CC_50_. Furthermore, increases of lysosomal volume of macrophages treated with EtOH-Ext, Hex-F, and Lupeol were observed. On the other hand, only Lupeol stimulated increase of phagocytic capability of macrophages, suggesting this compound might be characterized as the biomarker for the antileishmanial effect of* P. insignis* stem bark, as well as the involvement of immunomodulatory mechanisms in this effect.

## 1. Introduction

Leishmaniasis are parasitic diseases caused by protozoa from* Leishmania* genus. Humans are infected by the sting of sand flies which live in forest areas, caves, or rodent dens. This pathology is widely distributed throughout the world and occurs in Asia, Europe, Africa, and the Americas. Furthermore, there have been some reports of its occurrence in the American continent since colonial times [1, 2]. Leishmaniasis is capable of affecting both man and animals, affecting more than 12 million people worldwide, with 2 to 3 million of new cases each year [2, 3]. According to the World Health Organization (WHO), approximately 556 million people were in areas at risk of contamination of visceral leishmaniasis and 399 million for cutaneous leishmaniasis in 2014 [2].

Since the 1940s, chemotherapy of leishmaniasis has been based on the use of pentavalent antimonials as first choice drugs, such as sodium stibogluconate (Pentostan®) and meglumine antimoniate (Glucantime®) [4]. Second-line drugs, such as pentamidine and amphotericin B, are important in the therapy of patients with coinfections, or in cases of resistance to antimonials [5, 6]. However, these drugs require long-term parenteral administration and have serious adverse effects in addition to high relapse rates, especially in immunocompromised patients [5, 7]. Therefore, considering these difficulties, the search for new safer and more effective chemotherapeutic agents is markedly relevant.

The use of medicinal plants is a very promising alternative. Plants biosynthesize compounds for their own defense in response to an environmental attack [8]. Its importance is evidenced by the wide diversity of metabolites produced by these species, with distinct chemical, physical, and biological characteristics, most of them possibly bioactive against* Leishmania *sp. [9]. In this context, substances obtained from native species from Brazil which present antileishmanial activity and low toxicity may represent alternatives for the conventional treatment of leishmaniasis.

*Platonia insignis* Mart., popularly known as “bacurizeiro,” is a fruit and woody plant with dense and diversified population, occurring from the Amazon to Piauí in Brazil. It belongs to the Clusiaceae family, composed of 1000 species and 47 genera, distributed in tropical, subtropical, and temperate regions [10]. In Brazil, the use of* P. insignis* in traditional medical practices is based on its diverse biological properties, such as cicatrizant, antimicrobial, digestive, diuretic, antitumor, cytotoxic, and antioxidant [11].

Studies on the antileishmanial activity of medicinal plants represent a great challenge for the discovery and identification of new bioactive substances. Moreover, there are no studies regarding the antileishmanial potential of the extracts and fractions obtained from the stem bark of* P. insignis*. Therefore, this work aimed to assess the potential antileishmanial activity, cytotoxicity, and immunomodulatory effect of ethanol extract (EtOH-Ext), its hexanic fraction (Hex-F), and the main constituent Lupeol obtained from the stem bark of* P. insignis*.

## 2. Material and Methods

### 2.1. Chemicals

Dimethyl sulfoxide (DMSO: 99%), anhydrous sodium sulfate, glacial acetic acid, ethanol, formaldehyde, sodium chloride, calcium acetate, zymosan, and neutral red were purchased from Merck Chemical Company (Germany). Schneider's medium, RPMI 1640 medium, fetal bovine serum (FBS), MTT (3-(4,5-dimethylthiazol-2-yl)2,5-diphenyltetrazolium bromide), Griess reagent (1% sulfanilamide in H_3_PO_4_ 10% (v/v) in Milli-Q water), and the antibiotics penicillin and streptomycin were purchased from Sigma Chemical (St. Louis, MO, USA). The antibiotic amphotericin B (90%) was purchased from Cristália (São Paulo, SP, Brazil).

### 2.2. Plant Material

The stem bark of* P. insignis* was collected in September 2010, from the municipality of Timon, Maranhão, Brazil. The voucher specimen is deposited in the Herbarium Graziela Barroso of the Federal University of Piauí, Brazil, under number TEPB 20701.

### 2.3. Extraction of EtOH-Ext and Hex-F from Stem Bark of* P. insignis*

The stem bark of* P. insignis* was dried at room temperature and then powdered. The material obtained (1.1 kg) was exhaustively extracted six consecutive times with ethanol, during five days each using the following extraction procedure. Following removal of the solvent at low pressure in the rotary evaporator and lyophilization, the EtOH-Ext (114.5 g) was obtained. Afterwards, the EtOH-Ext extract (90.0 g) was suspended in a MeOH/H_2_O mixture (2 : 3), and subjected to the liquid-liquid partitioning process with hexane, resulting in the Hex-F (9.8 g; yield of 10.9%).

The EtOH-Ext and Hex-F were solubilized in DMSO to obtain stock solutions at 80 mg/mL. In each protocol, the stock solution was diluted in the appropriate culture media to reach the desired concentration, not exceeding the concentration of 0.5% DMSO.

### 2.4. Isolation of Triterpene Lupeol from Hex-F

The Hex-F (7.2 g) was subjected to column chromatography of silica gel (210 g), using hexane and ethyl acetate as eluent solvents. Fifty fractions were obtained. Similar fractions were then merged according to thin layer chromatography analysis. The group HB34 (fractions 34 to 36, 1370 mg, yield of 19.6%) was obtained after elution with hexane : AcOEt (95 : 5), and then analyzed by ^1^H NMR and ^13^C. The compound** 1** was identified as follows:

*Lupeol* ***(1)***. White amorphous solid. RMN ^1^H (400 MHz, CDCl_3_): *δ*1,67 (s, H-30), 3,18 (dd, *J* = 11.1, 5.1 Hz, H-3), 4,56 (*J* = 2.4 Hz, H-29a), 4,68 (*J* = 2.4 Hz, H-29b). RMN ^13^C (100 MHz, CDCl_3_): *δ* 38.8 (C-1), 27.6 (C-2), 79.1 (C-3), 39.0 (C-4), 55.4 (C-5), 18.4 (C-6), 34.4 (C-7), 41.0 (C-8), 50.6 (C-9), 37.3 (C-10), 21.1 (C-11), 25.3 (C-12), 38.2 (C-13), 42.9 (C-14), 27.5 (C-15), 35.7 (C-16), 43.1 (C-17), 48.4 (C-18), 48.1 (C-19), 151.0 (C-20), 30.0 (C-21), 40.1 (C-22), 28.1 (C-23), 15.5 (C-24), 16.2 (C-25), 16.1 (C-26), 14.7 (C-27), 18.1 (C-28), 109.5 (C-29), 19.4 (C-30).

### 2.5. *Leishmania* Culture Conditions

*Leishmania (Leishmania) amazonensis* (IFLA/BR/67/PH8) was maintained as amastigote forms by several weekly passages in BALB/c mice, and as promastigote forms at 26°C in supplemented Schneider's medium, pH 7.0 (10% heat-inactivated fetal bovine serum (FBS), 100 U/mL penicillin, and 100 *µ*g/mL streptomycin), as previously described [12]. Extracellular amastigote-like forms were obtained by* in vitro* differentiation of promastigotes of* Leishmania amazonensis* in the stationary growth phase by increasing the temperature to 32°C and decreasing the pH to 4.6 [13].

### 2.6. Animals and Murine Peritoneal Macrophages

Male and female BALB/c mice (4-5 weeks old) obtained from Section of Biotherium from Medicinal Plants Research Center of Federal University of Piauí, (Teresina, Piauí, Brazil), were maintained under controlled temperature (24 ± 1°C) and light conditions (12-h light/dark cycle). Inflammatory peritoneal macrophages were collected 5 days after intraperitoneal administration with 1.5 mL of 3% thioglycollate medium. Animals were handled according to the Resolution number 1000/2012 (Federal Council of Veterinary Medicine, Brazil) for the Care and Use of Animals. All experimental protocols were approved by the Research Ethics Committee (CEEA-UFPI number 014/2015).

### 2.7. Bioassay of Promastigote and Axenic Amastigote Forms of* Leishmania amazonensis*

Promastigote and axenic amastigote forms were seeded in 96-well plates containing Schneider's supplemented medium (1 × 10^6^* Leishmania* per 100 *μ*l medium). Then, EtOH-Ext, Hex-F, and Lupeol were added in triplicate at the concentrations of 6.25, 12.5, 25, 50, 100, 200, 400, and 800 *µ*g/mL. Amphotericin B (Amph B, 2 *µ*g/mL) was used as positive control. The plate was maintained in a biological oxygen demand (BOD) incubator at a temperature of 26°C for 48 h. Six hours before the end of this period, resazurin (1 × 10^−3^ mol/L, 20 *µ*L) was added in each well. All the absorbance was read using a Biotek microplate reader (model ELx800) at 550 nm. The results were expressed as percent inhibition in growth [14].

### 2.8. Investigation of EtOH-Ext, Hex-F, and Lupeol Activities on* L. amazonensis*-Infected Macrophages

Murine peritoneal macrophages were harvested and plated on a 24-well microplate (1 × 10^6^ cells/mL) in supplemented RPMI 1640 (10% inactivated FBS, 100 U/ml penicillin, and 100 *μ*g/mL streptomycin), containing sterile 13 mm round coverslips. Culture plates were incubated at 37°C and 5% CO_2_ for 3 h for cell adhesion. Adhered macrophages were then incubated with a new medium containing promastigotes (in stationary phase) at a ratio of ten promastigotes to one macrophage at 5% CO_2_ and 37°C for 4 h. Afterwards, the cells were washed with 0.01 M phosphate buffered saline (PBS). Then, the EtOH-Ext, Hex-F, and Lupeol were incubated for 48 h at concentrations related to the IC_50_, 1/2 IC_50_, and 1/4 IC_50_ of each treatment against promastigote forms, as well as Amph B (2 *µ*g/mL) as positive control. After this period, the coverslips were removed and stained with Panoptic staining kit. For each treatment, the number of infected macrophages and the parasite load were counted using optical microscopy [12].

### 2.9. Cytotoxic Effect on Macrophages and Determination of Selectivity Index (SI)

Cytotoxicity evaluation was carried out in 96-well plates using the MTT assay. Approximately 1 × 10^6^ murine peritoneal macrophages per well were incubated in 100 *μ*L of supplemented RPMI 1640 medium at 37°C and 5% CO_2_ for 4 h for cell adhesion. Nonadherent cells were removed by washing with RPMI 1640 medium. Then, EtOH-Ext, Hex-F, and Lupeol were diluted in supplemented RPMI 1640 medium, added at concentrations of 6.25, 12.5, 25, 50, 100, 200, 400, and 800 *µ*g/mL, and incubated at 37°C with 5% CO_2_ for two days. Following incubation, cytotoxicity was assessed by adding MTT (5 mg/mL). The supernatant was discarded and the formazan crystals were dissolved in 100 *μ*L of DMSO. Finally, absorbance was measured at 550 nm using Biotek microplate reader (model ELx800). Selectivity index of each treatment was calculated at the ratio of mean cytotoxicity concentration (CC_50_) for BALB/c mice murine peritoneal macrophages and half-maximal inhibitory concentration (CI_50_) for macrophage-internalized amastigote forms of* L. amazonensis* [15].

### 2.10. Evaluation of Immunomodulatory Activity

#### 2.10.1. Solutions

The stock solution of neutral red (NR) dye was prepared by solubilizing 0.002 g in 1 mL of DMSO. The extraction solution used in protocols described in Sections 2.10.2 and 2.10.3 consisted of 96% glacial acetic acid (1.0%) and ethanol (50%) dissolved in deionized water (v/v). The staining of macrophages for the phagocytic capability assay was obtained by diluting 0.3 mL of the neutral red solution and 0.02 g of zymosan in 3 mL of PBS. The Baker's fixative solution was composed of 4% formaldehyde (v/v), 2% sodium chloride (w/v), and 1% calcium acetate (w/v) in deionized water. Griess' reagent was prepared with 1.0% sulfanilamide in 10% H_3_PO_4_ (v/v) and 0.1% naphtylenediamine in Milli-Q® water.

#### 2.10.2. Lysosomal Volume

The uptake of the cationic dye NR from lysosomal compartments of macrophages was assessed as previously described [16]. Murine peritoneal macrophages (1 × 10^6^ cells per well) were harvested and plated with EtOH-Ext, Hex-F, and Lupeol in serial dilutions at 37°C and 5% CO_2_ for 48 h. Next, cells were treated with 10 *µ*L of 2% neutral red solution (see Section 2.10.1) and incubated for 30 min. The wells were subsequently washed twice with PBS, and the internalized neutral red was solubilized by adding the extraction solution (see Section 2.10.1). After 30 min on a Kline shaker (model AK 0506), the absorbance at 550 nm was measured as above.

#### 2.10.3. Phagocytosis Capability

Murine peritoneal macrophages (1 × 10^6^ cells per well) were harvested and plated with EtOH-Ext, Hex-F, and Lupeol in serial dilutions at 37°C and 5% CO_2_ for 48 h. After incubation, 10 *µ*L of NR-stained zymosan solution (described above) was added and the cells were incubated at 37°C for 30 min. Following incubation, 100 *µ*L of Baker's fixative solution (see Section 2.10.1) was added in order to stop the phagocytosis of zymosan. After 30 min, the cells were washed with PBS to remove nonphagocytosed NR and zymosan. Then, NR was solubilized by adding the extraction solution (see Section 2.10.1) in the Kline shaker. The absorbance was subsequently measured at 550 nm as above [17].

#### 2.10.4. Nitrite Measurement

Murine peritoneal macrophages (2 × 10^5^ cells per well) were harvested and plated with EtOH-Ext, Hex-F, and Lupeol in serial dilutions at 37°C and 5% CO_2_ for 24 h. Then, the supernatants (100 *µ*L) were incubated with equal parts of Griess' reagent (see Section 2.10.1), and the absorbance was read at 550 nm as above. The standard curve was prepared with sodium nitrite in Milli-Q water at concentrations of 1, 5, 10, 25, 50, 75, 100, and 150 *μ*M diluted in RPMI 1640 medium [18].

### 2.11. Statistical Analysis

All assays were performed in triplicate in three independent experiments. The mean inhibitory concentration (IC_50_) and mean cytotoxic concentration (CC_50_) were calculated using probit regression with 95% confidence limit using SPSS software version 13.0. One-way analyses of variance (ANOVA) followed by Bonferroni's post hoc test were performed using the GraphPad Prism software version 5.0. Differences were considered statistically significant when *P* < 0.05.

## 3. Results

### 3.1. Identification of Lupeol in Hex-F

**Figure 1:**
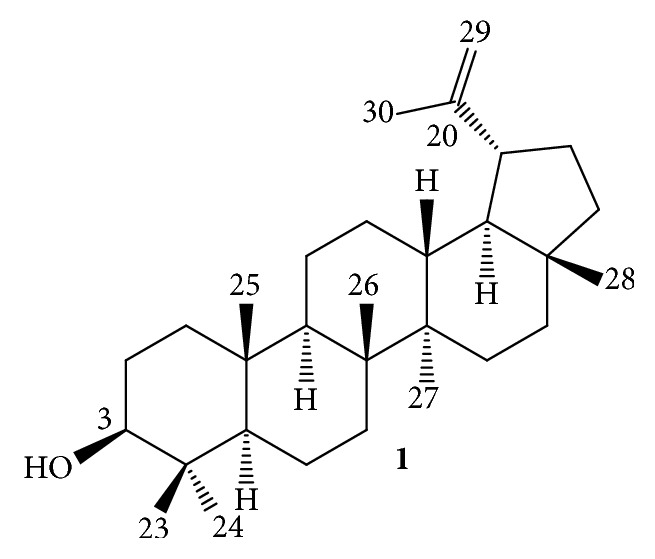
The triterpene Lupeol.

**Figure 2:**
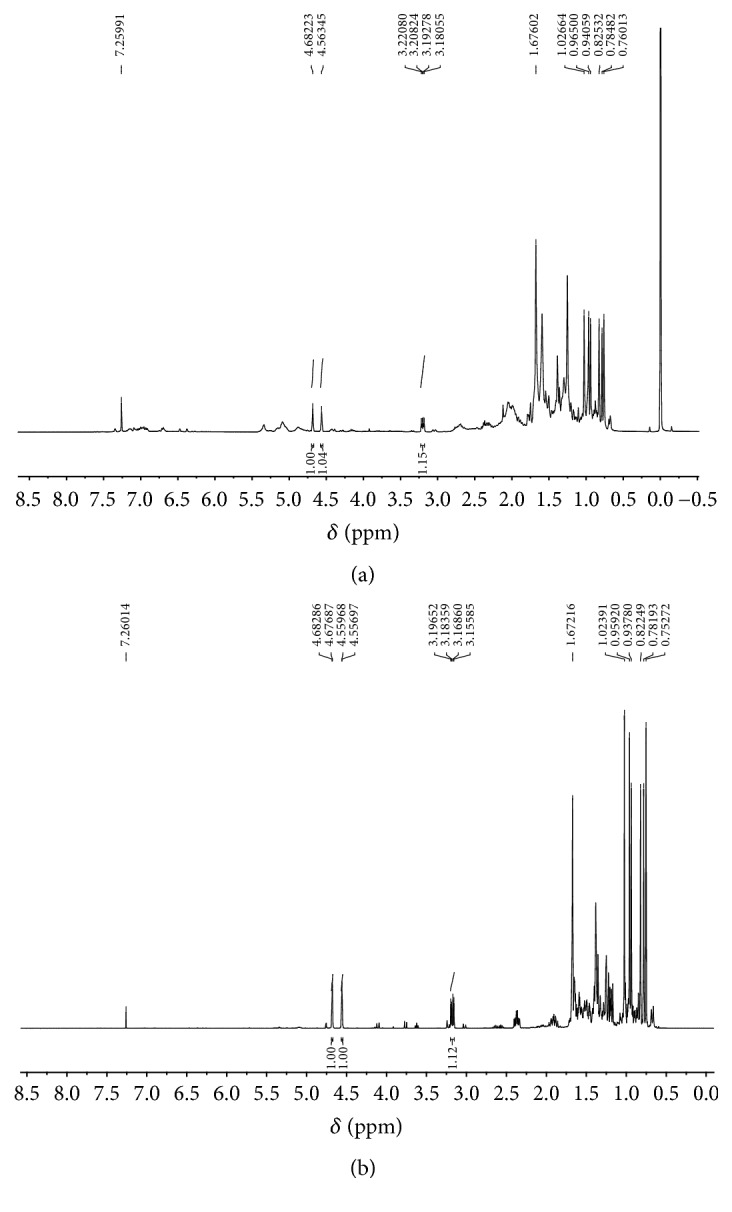
^1^H NMR spectra (CDCl_3_, 400 MHz): Hex-F (a) and Lupeol (b).

The hexane fraction from* P. insignis* (Hex-F) provides Lupeol as the major compound (Figure 1), confirmed by comparison between ^1^H NMR spectra of the Hex-F and the isolated compound (Figure 2). The Lupeol was identified after isolation and analysis by ^1^H and ^13^C NMR when compared with data previously reported [19].

### 3.2. Anti-*Leishmania* Activity Assay

**Figure 3:**
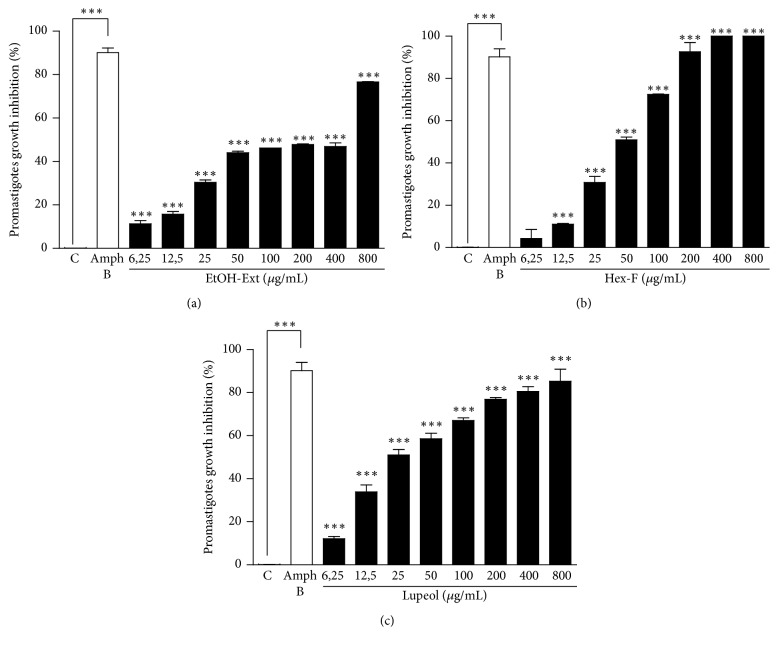
Anti-*Leishmania* activity of EtOH-Ext (a), Hex-F (b), and Lupeol (c) from* P. insignis* stem bark against promastigote forms of* L. amazonensis*. Parasites (1 × 10^6^) were exposed to different concentrations for 48 h, and cell viability was assessed by resazurin assay. ^*∗∗∗*^*P* < 0.001 versus control.

The EtOH-Ext promoted statistically significant inhibition of* L. amazonensis* promastigotes forms at all concentrations tested (Figure 3(a)). Besides, the Hex-F showed growth inhibition ranging from the concentration of 12.5 *µ*g/mL to 400 *µ*g/mL, where 100% of inhibition was achieved (Figure 3(b)). Lupeol was the most effective in promoting growth inhibition of promastigotes, with significant inhibition at all concentrations tested (Figure 3(c)). The mean inhibitory concentrations for EtOH-Ext, Hex-F, and Lupeol against promastigote forms were calculated and listed in Table 1.

**Figure 4:**
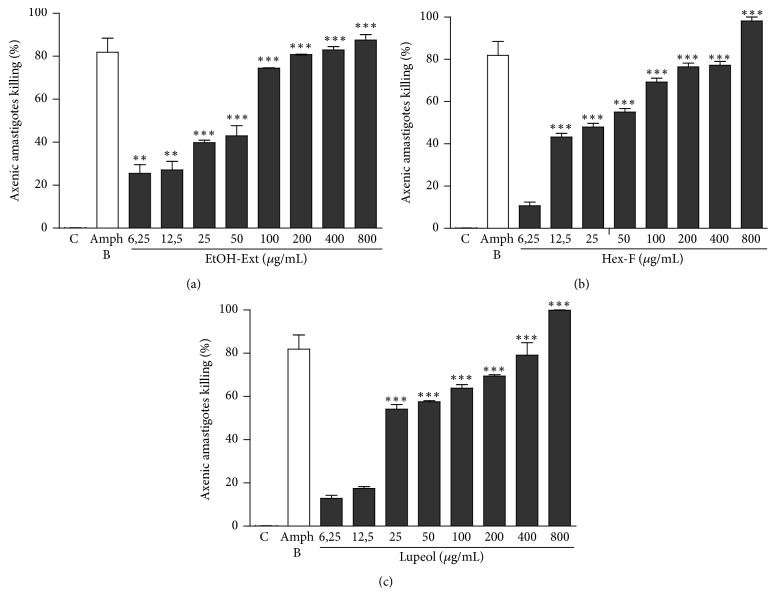
Anti-*Leishmania* activity of EtOH-Ext (a), Hex-F (b), and Lupeol (c) against axenic amastigote forms of* L. amazonensis*. Parasites (1 × 10^6^) were exposed to different concentrations for 48 h, and cell viability was assessed by resazurin assay. ^*∗∗*^*P* < 0.01 versus control; ^*∗∗∗*^*P* < 0.001 versus control.

In another set of experiments, the effects of EtOH-Ext, Hex-F, and Lupeol on axenic amastigote forms of* L. amazonensis *were assessed. The EtOH-Ext exhibited a significant inhibitory effect starting from the lowest concentration tested (Figure 4(a)), whereas the inhibitory effect of Hex-F started at concentration of 12.5 *µ*g/mL, reaching 100% of inhibition at the highest concentration tested (Figure 4(b)). The Lupeol presented significant anti-*Leishmania* activity when compared to control started at concentration of 25 *µ*g/mL (Figure 4(c)). The mean inhibitory concentrations (IC_50_) for EtOH-Ext, Hex-F, and Lupeol against axenic amastigote forms were calculated and listed in Table 1.

### 3.3. Cytotoxicity Determination

**Figure 5:**
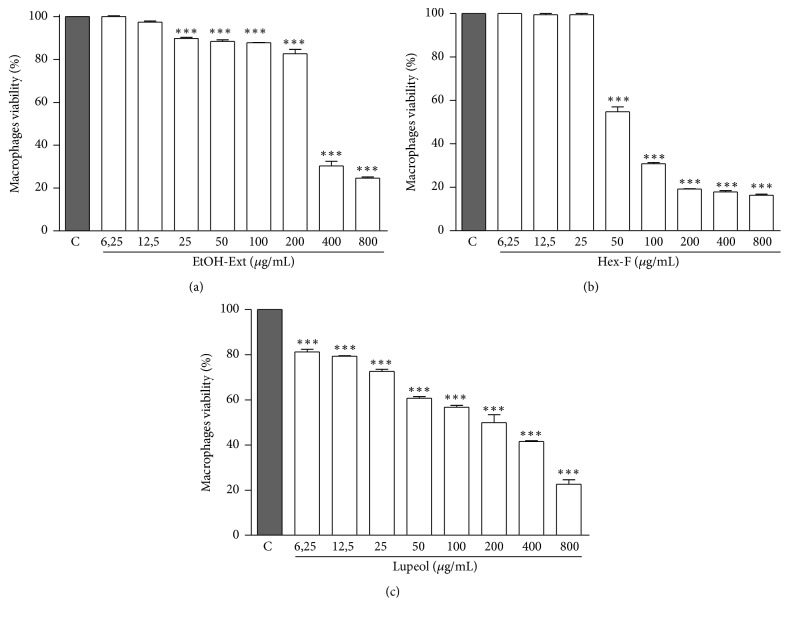
Cytotoxic effects against murine peritoneal macrophages of EtOH- Ext (a), Hex-F (b), and Lupeol (c). Murine peritoneal macrophages were incubated for 48 h in the presence of different concentrations. Macrophage viability was measured by MTT assay (MTT). Data are expressed as means ± SEM of three experiments performed in triplicate. ^*∗∗∗*^*P* < 0.001 versus control.

The EtOH-Ext demonstrated significant cytotoxicity starting from the concentration of 25 *μ*g/mL, presenting higher cytotoxic effect at concentrations of 400 and 800 *μ*g/mL (Figure 5(a)). Thereafter, the Selectivity index (SI) was calculated by the ratio of mean cytotoxic concentration (CC_50_) and mean inhibitory concentration (IC_50_) for axenic amastigote forms of* L. amazonensis*. The EtOH-Ext presented a selectivity index of 8.42, indicating the EtOH-Ext is around 8-fold more cytotoxic to the parasite than to the host cells.

The Hex-F showed significant cytotoxicity (Figure 5(b)) by altering the viability of the cells starting from the concentration of 50 *μ*g/mL, with the maximal cytotoxic effect at 200 *μ*g/mL, and SI of 1.99 (Table 1). On the other hand, the main constituent Lupeol also significantly affected the viability of macrophages (Figure 5(c)), with CC_50_ and SI higher than those obtained for Hex-F. These results indicate less cytotoxicity to the host cells with similar efficacy against the parasite (Table 1).

### 3.4. Effect on Macrophage Infection

**Figure 6:**
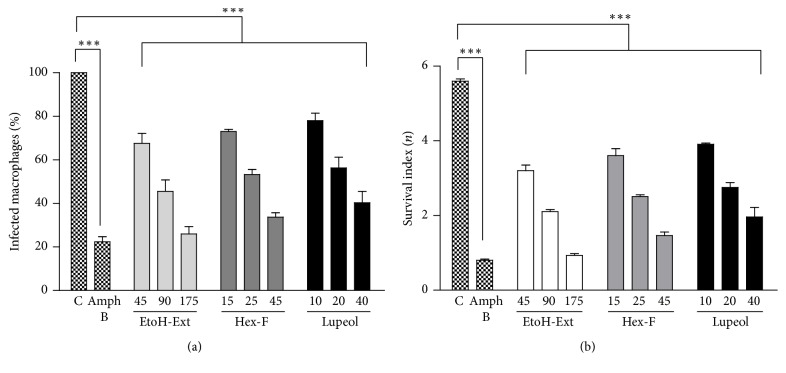
Effect of EtOH-Ext, Hex-F, Lupeol, and the reference drug Amph B (2 *μ*g/mL) on the infected macrophages (a) and the survival index of macrophage-internalized amastigotes (b) after 48 h of exposure. Murine peritoneal macrophages were infected with promastigote forms of* L. amazonensis* and then treated with concentrations related to respective IC_50_, 1/2 IC_50_, and 1/4 IC_50_ against promastigotes. Results represent the means ± SEM of three experiments performed in triplicate. ^*∗∗∗*^*P* < 0.001 versus control.

The effects of the treatment with EtOH-Ext, Hex-F, and Lupeol of macrophages infected with* L. amazonensis* are shown in Figure 6. They were able to significantly reduce the number of infected macrophages in all tested concentrations when compared to the control (Figure 6(a)). Furthermore, a significant decrease of number of internalized amastigotes was observed in all tested concentrations of EtOH-Ext, Hex-F, and Lupeol when compared with control group (Figure 6(b)).

### 3.5. Lysosomal Volume and Phagocytosis Test

**Figure 7:**
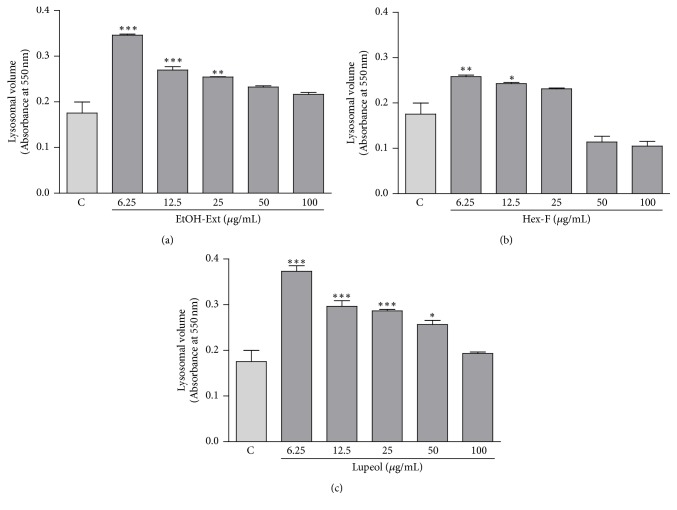
The influence of EtOH- Ext (a), Hexanic fraction (b), and Lupeol (c) on the lysosomal volume in macrophages. Murine peritoneal macrophages were incubated for 48 h after treatment. Lysosomal volume was analyzed spectrophotometrically by the increase in neutral red (NR) uptake following solubilization with the extraction solution. Data are presented as mean ± SEM of three experiments carried out in triplicate. ^*∗*^*P* < 0.05 versus control; ^*∗∗*^*P* < 0.01 versus control*; *^*∗∗∗*^*P* < 0.001 versus control.

The lysosomal volume was assessed by the retention of the NR the lysosome of macrophages and then determined colorimetrically. The EtOH-Ext induced a significant increase of lysosomal volume of macrophages at concentrations of 6.25, 12.5, and 25 *μ*g/mL (Figure 7(a)). Besides, the lysosomal volume after treatment with Hex-F increased significantly at concentrations of 6.25 and 12 *μ*g/mL (Figure 7(b)). Interestingly, the lysosomal volume of macrophages treated with Lupeol showed a statistically significant increase of the endocytic volume at concentrations ranging from 6.25 to 50 *μ*g/mL of Lupeol (Figure 7(c)).

**Figure 8:**
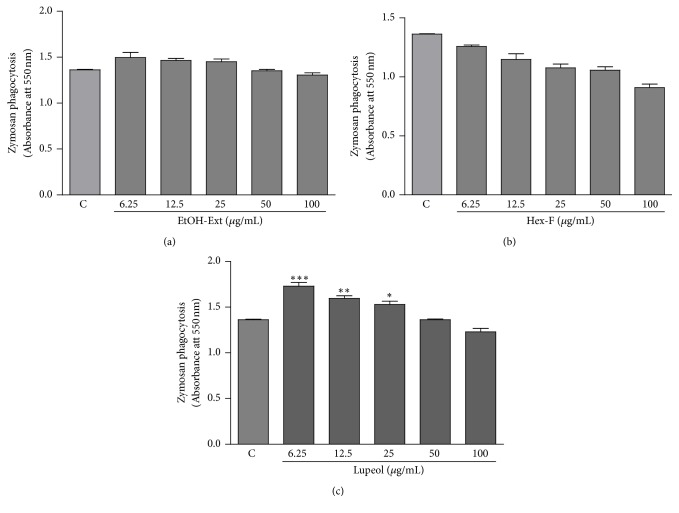
The influence of EtOH- Ext (a), Hexanic fraction (b), and Lupeol (c) on the phagocytosis in macrophages. Murine peritoneal macrophages were treated with a range of concentrations for 48 h. The phagocytosis was analyzed by incorporation of NR-stained zymosan, solubilized with the extraction solution. Data are presented as mean ± SEM of three experiments carried out in triplicate. ^*∗*^*P* < 0.05 versus control; ^*∗∗*^*P* < 0.01 versus control*; *^*∗∗∗*^*P* < 0.001 versus control.

Other parameter related to macrophages activation parameter was the phagocytic capability of stained zymosan particles. Both EtOH-Ext and Hex-F were not able to promote any increase in the phagocytic capability of macrophages (Figures 8(a) and 8(b)). Interestingly, a significant increase in phagocytosis of zymosan particles was observed in macrophages treated with Lupeol at concentrations ranging from 6.25 to 25.0 *μ*g/mL (Figure 8(c)).

### 3.6. Measurement of Nitrite Production

**Figure 9:**
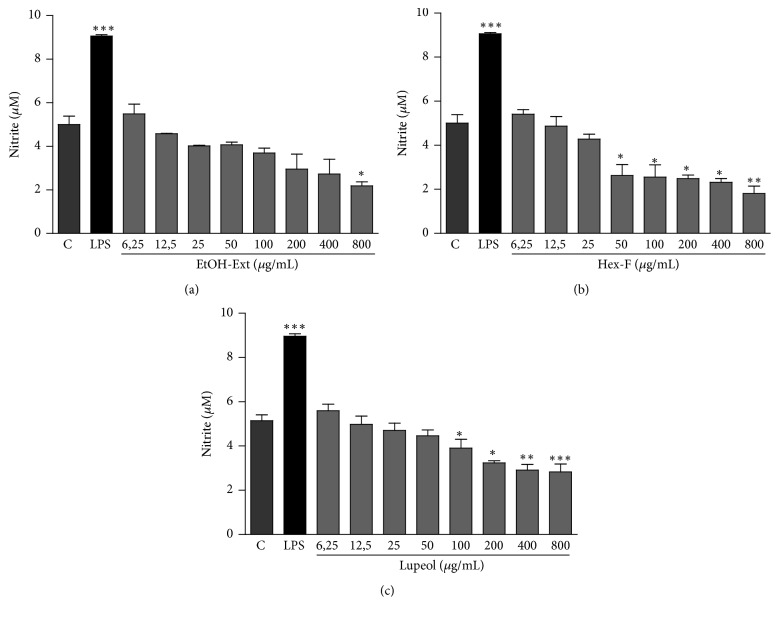
Production of nitrite in murine peritoneal macrophages treated with EtOH-Ext (a), Hex-F (b), and Lupeol (c). Murine peritoneal macrophages were treated and then incubated for 24 h. After this period, supernatants were mixed with Griess' reagent in equal parts. LPS, bacterial lipopolysaccharide (2 mg/mL). Data are presented as mean ± SEM of three experiments carried out in triplicate. ^*∗*^*P* < 0.05 versus control; ^*∗∗*^*P* < 0.01 versus control*; *^*∗∗∗*^*P* < 0.001 versus control.

The nitrite measurement was determined in macrophages after incubation with EtOH-Ext, Hex-F, and Lupeol. No increase in nitrite production was observed after all treatments. Furthermore, a decrease in nitrite production was observed only at concentrations higher than those observed for lysosomal volume and phagocytic capability (Figure 9).

## 4. Discussion

Scientific studies related to the pharmacological potential of medicinal plants have been increasing. The use of medicinal plants in traditional medical practices has increasingly proved that stems, roots, leaves, seeds, and fruits of these plants have appreciable efficacy in the cure of several diseases. Previous studies have demonstrated potent antileishmanial activity of extracts and fractions from leaves and fruit peels of* Azadirachta indica* with IC_50_ value of 1.1 *μ*g/mL against promastigote forms, and 0.4 *μ*g/mL against amastigote forms, as well as low cytotoxicity against mammalian cells [12]. Furthermore, the essential oil from leaves of* Eugenia uniflora *demonstrated a marked antileishmanial effect with IC_50_ of 1.75 *μ*g/mL against promastigotes, and 1.92 *μ*g/mL against amastigotes [15]. Thus, the observation of the therapeutic properties of natural products has led towards the research of the active principles from several plant species [15, 20].

The* P. insignis* species, called “bacuri” provides a very popular consumed fruit among the Brazilian Amazonian people. This ethnopharmacological use is related to the seeds extract as cicatrizant and anti-inflammatory agents. Currently, several activities have been reported for all parts of this fruit (seed, shells, and pulp). Besides, classes of terpenes, xanthones, and phenolics have been reported as major constituents [21]. The Hex-F obtained from the stem bark of* P. insignis* presents the triterpene Lupeol as its major constituent. Triterpenes have aroused a marked interest due to their broad spectrum of biological activities [22]. In this context, the triterpene Lupeol has demonstrated potential application in studies concerning the discovery of compounds with antitumor and anti-inflammatory activities [23].

The screening of leishmanicidal agents classically uses promastigotes forms due to their simplicity and low cost of the culture [24]. Moreover, Costa-Júnior and colaborators [25] have reported the antileishmanial effect of garcinielliptone FC, a polyisoprenilated benzophenone isolated from the seed extracts of* P. insignis*, with a IC_50_ value of 25.78 *µ*g/mL. This evidence reinforces the anti-*Leishmania* potential of these plant species. In this context, EtOH-Ext, Hex-F, and Lupeol demonstrated* in vitro* efficacy against both promastigotes and axenic amastigotes of* L. amazonensis*. In this study, no significant difference was observed between IC_50_ values obtained for Lupeol and Hex-F, then indicating the Hex-F-induced antileishmanial effect might be related to its Lupeol content. Thus, the triterpene Lupeol can be characterized as a biomarker for the antileishmanial activity of* P. insignis*.

Previous studies have suggested that lipophilic compounds, such as triterpenes, act by a peculiar mechanism. These compounds can pass easily through the cytoplasmic membranes, affecting structures of their different layers of polysaccharides, fatty acids, and phospholipids, thus making them permeable [26]. Once they cross the membrane, the coagulation of cytoplasm can occur. These events are able to promote the interruption of specific metabolic pathways of lipids and proteins [27], interference in cell division [28, 29], or stimulate the depolarization of the mitochondrial membranes, which can lead the cell to trigger necrosis or apoptosis mechanisms [30].

Considering that EtOH-Ext, Hex-F, and Lupeol showed significant antileishmanial activity, the investigation of the cytotoxic activity against mammalian cells as potential hosts is markedly necessary. Cytotoxicity tests against murine peritoneal macrophages were performed by the resazurin colorimetric assay [31]. In these experiments, CC_50_ values higher than the IC_50_ values for promastigotes and axenic amastigotes were found. Conversely, previous studies have found a SI lower than 1.0 for meglumine antimoniate, the reference drug for treatment of leishmaniasis, demonstrating a higher cytotoxicity against macrophages than the parasite. This result is related to the* in vivo *effects of pentavalent antimonials, where, despite being first-line drugs for the treatment of cutaneous and visceral leishmaniasis, numerous side effects and toxicity have been reported [32]. Therefore, EtOH-Ext, Hex-F, and Lupeol present SI higher than 1.0, which reveals the safety of these substances and the continuity in further studies of antileishmanial activity.

Once the EtOH-Ext, Hex-F, and the main constituent Lupeol showed effective antileishmanial activity with selective action towards the parasite, we decided to investigate their activities against the intramacrophagic amastigotes. This experimental model assesses the effects against the parasite by the activation of microbicidal mechanisms in the host macrophages and therefore is as closest as possible to the* in vivo* effect [33]. In these experiments, all treatments presented antileishmanial activity against the macrophage-internalized amastigotes, which was observed by the significant reduction in the number of infected macrophages and in the number of amastigotes per macrophage. Thus, these results reinforce studies concerning which mechanisms triggered in macrophages might be involved in this effect.

Activated macrophages perform several functions, including phagocytosis, increased numbers of hydrolytic enzymes from lysosomal compartments, tumor cytotoxicity, cytokine secretion, and antigen presentation. Phagocytosis and lysosomal volume are functions of innate immunity, important for the control of infections and leading to the degradation of pathogens and the presentation of antigens [34, 35]. Thus, in order to evaluate this hypothesis, parameters related to activation of macrophages and structural mechanisms of antileishmanial activity (lysosomal compartment volume and phagocytic activity), as well as the nitrite production as a mechanism of the activation state of the macrophages, were assessed.

The incorporation of neutral red into the secretory vesicles was applied to study the lysosomal volume. Besides, the phagocytic capability was determined by the incorporation of zymosan particles stained with neutral red. Zymosan is a polysaccharide from the cell wall of* Saccharomyces cerevisiae* in the form of water-insoluble powder [36]. In this study, the antiamastigote activity of EtOH-Ext, Hex-F, and Lupeol may involve the increase of lysosomal volume, since all the treatments promoted an increase of the volume of the endocytic compartment. Interestingly, the increase of phagocytic capacity induced by Lupeol may also be related to its antiamastigote activity, considering Lupeol was the only treatment which increase the phagocytosis of zymosan particles in macrophages.

Another important pathway that may be involved in the mechanism of antileishmanial activity is stimulation of NO production by macrophages. This mechanism has been considered as the most effective mechanism underlying defense against* Leishmania* spp. infection [37]. This production can be stimulated* in vitro* by the activation of macrophages using lipopolysaccharide (LPS), which activates the inducible nitric oxide synthase (iNOS), then leading to the production of high concentrations of NO. However, the results showed that EtOH-Ext, Hex-F, and Lupeol lack the ability to stimulate NO production by macrophages, indirectly determined by the nitrite measurement using Griess' Reaction. Thus, the antiamastigote effect of these treatments probably does not involve the NO pathway.

## 5. Conclusion

This study demonstrates that EtOH-Ext, Hex-F, and Lupeol obtained from* P. insignis* were able to promote effective and selective antileishmanial activity against both promastigote and amastigote forms of* L. amazonensis*, as well as against macrophage-internalized amastigote forms. This study also demonstrated the mechanisms underlying antileishmanial effect, such as the increase of lysosomal volume of macrophages for all treatments. Interestingly, only Lupeol was able to promote increase the phagocytic capacity of macrophages, suggesting this compound might be characterized as the biomarker for the antileishmanial effect of* P. insignis* stem bark. Thus, the involvement of innate immune system was evidenced. Further investigations are recommended in order to determine the potential antileishmanial effect of this species in experimental* in vivo* models of leishmaniasis.

## Figures and Tables

**Table 1:** Anti-*Leishmania* activity of EtOH-Ext, Hex-F, and Lupeol from *P. insignis *stem bark, and cytotoxic effects against murine peritoneal macrophages.

Compounds	Macrophages	Promastigotes	Axenic amastigotes	
	CC_50_ (*µ*g/mL)	CI_50_ (*µ*g/mL)	CI_50_ (*µ*g/mL)	SI
EtOH-Ext	341.95	174.24	40.58	8.42
Hex-F	71.65	45.23	35.87	1.99
Lupeol	144.00	39.06	44.10	3.26
Amph B	8.75	1.74	0.20	43.75

SI: Selectivity index for axenic amastigote forms of *L. amazonensis* (CC_50_/IC_50_).
